# Lipocalin-2 Functions as Inhibitor of Innate Resistance to *Mycobacterium tuberculosis*

**DOI:** 10.3389/fimmu.2018.02717

**Published:** 2018-11-26

**Authors:** Sara Louise Dahl, Joshua S. Woodworth, Christian Johann Lerche, Elisabeth Præstekjær Cramer, Pia Rude Nielsen, Claus Moser, Allan Randrup Thomsen, Niels Borregaard, Jack Bernard Cowland

**Affiliations:** ^1^Granulocyte Research Laboratory, Rigshospitalet, Copenhagen, Denmark; ^2^Department of Infectious Disease Immunology, Statens Serum Institute, Copenhagen, Denmark; ^3^Department of Clinical Microbiology, Rigshospitalet, Copenhagen, Denmark; ^4^Department of Pathology, Zealand University Hospital, Roskilde, Denmark; ^5^Department of Immunology and Microbiology, University of Copenhagen, Copenhagen, Denmark; ^6^Department of Clinical Genetics, Rigshospitalet, Copenhagen, Denmark

**Keywords:** *Mycobacterium tuberculosis*, NGAL, 24p3, iron metabolism, innate immunity, neutrophils, Lcn2

## Abstract

Lipocalin-2 is a constituent of the neutrophil secondary granules and is expressed *de novo* by macrophages and epithelium in response to inflammation. Lipocalin-2 acts in a bacteriostatic fashion by binding iron-loaded siderophores required for bacterial growth. *Mycobacterium tuberculosis* (M.tb) produces siderophores that can be bound by lipocalin-2. The impact of lipocalin-2 in the innate immune response toward extracellular bacteria has been established whereas the effect on intracellular bacteria, such as M.tb, is less well-described. Here we show that lipocalin-2 surprisingly confers a growth advantage on M.tb in the early stages of infection (3 weeks post-challenge). Using mixed bone marrow chimeras, we demonstrate that lipocalin-2 derived from granulocytes, but not from epithelia and macrophages, leads to increased susceptibility to M.tb infection. In contrast, lipocalin-2 is not observed to promote mycobacterial growth at later stages of M.tb infection. We demonstrate co-localization of granulocytes and mycobacteria within the nascent granulomas at week 3 post-challenge, but not in the consolidated granulomas at week 5. We hypothesize that neutrophil-derived lipocalin-2 acts to supply a source of iron to M.tb in infected macrophages within the immature granuloma, thereby facilitating mycobacterial growth.

## Introduction

Adaptive immunity against *Mycobacterium tuberculosis* (M.tb) is well-described, whereas the role of the innate immune response that acts at the early stages of infection is not fully defined. Lipocalin-2 is an innate immune protein, also known as neutrophil gelatinase-associated lipocalin (NGAL), originally identified as a major constituent of the neutrophil secondary granules (1, 2). In neutrophils, lipocalin-2 is produced exclusively at the myelocyte and metamyelocyte stages of granulopoiesis in the bone marrow and stored in the secondary granules (3). Following the release of neutrophils from bone marrow, lipocalin-2 can be exocytosed from the pre-formed granules. Later, it has been recognized that lipocalin-2 can also be synthesized *de novo* by epithelial cells and macrophages in response to inflammatory stimuli (4–7). In mice (8, 9), but not in humans (10), lipocalin-2 is also produced in the liver as an acute phase-protein.

The best described function of lipocalin-2 is the capacity to prevent growth and spread of microorganisms that require siderophore-mediated uptake of iron (11). Siderophores are produced by a range of microorganisms, including M.tb (12), and bind ferric iron with high affinity by scavenging the host's iron deposits (13). Lipocalin-2 forms a ternary structure with the siderophore preventing bacterial uptake of siderophore-bound iron thus acting bacteriostatically (11). Lipocalin-2 knock-out mice (*Lcn2*^−/−^ mice) are more susceptible to infection by siderophore-producing pathogens, such as *Klebsiella pneumonia* (14, 15) and *Escherichia coli* (16, 17).

Pathogenic mycobacteria produce two types of siderophores that can be bound by lipocalin-2: mycobactin, an envelope-restricted siderophore, and the water soluble carboxymycobactin. Both siderophores bind Fe^3+^ with high affinity and sequester soluble as well as protein-bound iron (18–20). Only a few studies have investigated the *in vivo* role of lipocalin-2 in mycobacterial infection models (21–23) and the focus has been on the role of lipocalin-2 at late time points following infection when the adaptive immune response is activated and mature granulomas have been formed. However, since lipocalin-2 is produced in cells that are primarily active in the early innate immune response, we wanted to address the role of lipocalin-2 in control of M.tb infection before the transition of the local inflammatory environment to an adaptive immune response associated with recruitment of antigen-specific T cells. Furthermore, we wanted to elucidate whether the cellular origin of lipocalin-2 is important for infection control by establishing a chimeric bone marrow model that would allow us to discriminate myeloid cell-derived lipocalin-2 from epithelia cell-derived lipocalin-2. In this study, we report for the first time that lipocalin-2 promote M.tb replication early in infection. Moreover, we find that the cellular origin of lipocalin-2 is important in M.tb infection, since only neutrophil-derived lipocalin-2 is associated with an increased susceptibility to M.tb infection in this model. The increased susceptibility to M.tb infection mediated by neutrophil-derived lipocalin-2 could be related to the close proximity of neutrophils and M.tb within the immature granuloma.

## Methods

### Mice

B6.*Lcn2*^−^^/–^ mice (16) and C57BL/6NTac wildtype (WT) mice were bred at Taconic, Denmark. Congenic B6.SJL-*Ptprc*^*a*^*Pepc*^*b*^/BoyCrl (SJL.WT) wildtype mice, used in the transplantation setup, were bred at Charles River's facility in Italy. All mice were bred in opportunist-free barriers. M.tb infected mice were housed in a biosafety level III facility in ventilated cages with laminar air flow and with water and food *ad libitum*. All mice used in the experiments were females between 8 and 12 weeks old.

### Bone marrow transplantation

The congenic CD45 (Ly-5) mouse system was employed wherein SJL.WT mice expressing the CD45.1 variant of the leukocyte surface marker CD45, and WT and *Lcn2*^−^^/–^ mice expressing the CD45.2 variant, were used in the transplantation experiment. Mice were lethally irradiated with 900 cGy followed by transplantation with bone marrow cells from donor mice by intravenously injection into the tail vein of recipient mice 1 day after lethal irradiation. All recipients received 5 × 10^6^ bone marrow cells. Mice were housed in individually ventilated cages and handled under restricted hygienic conditions. Ciprofloxacin (Actavis, 100 mg/L) was added to their drinking water for the first 21 days. Seven weeks post-transplantation neutrophil chimerism was determined on jaw vein blood from all mice included in the transplantation setup. Macrophage chimerism was evaluated in a pilot study on bronchoalveolar lavage (BAL fluid). Antibodies used for determining chimerism were: Rat anti-mouse Gr-1 APC (1:300, clone RB6-8C5, BioLegend), rat anti-mouse F4/80 (1:100, clone BM8, BioLegend) and mouse anti mouse CD45.1 PE (1:50, clone A20, eBioscience), and CD45.2 FITC (1:200, clone 104, BD).

### Mycobacterial infection

For all experiments M.tb strain H37Rv was used. H37Rv was grown in Middlebrook 7H9 broth with glycerol (BD, 221832) supplemented with ADC enrichment (BD, 211887). Mid log phase cultures were aliquoted and stored at −80°C.

Mice were challenged with M.tb H37Rv using Biaera AeroMP system. Briefly, an H37Rv aliquot was thawed and the suspension sonicated for 5 min in a water-bath and drawn three times through a 27 gauge needle to ensure a proper dispersion of mycobacteria. Mycobacteria were diluted in 15 ml phosphate-buffered saline and delivered by aerosol route ~1,000 colony forming units (CFU) per mouse (as determined by total lung CFU 1 day after challenge). Aerosol suspensions were serially diluted and plated to confirm consistent exposure between experiments.

Mice were sacrificed at the time points indicated in the individual experiments subsequent to scoring on a scale from 0 to 5 based on the following clinical criteria: 0: Mouse is unaffected, 1: Mouse is slightly affected (e.g., slight piloerection or slightly commencing waistline), 2: Mouse is affected (e.g., piloerection, commencing waistline, and slightly decreased mobility), 3: The mouse is obviously affected (e.g., closed eyes, marked waistline, hunch-backed posture, affected breathing, decreased mobility), 4: Mouse is severely affected and should be euthanized, 5: The mouse is dead.

Mice were sedated with Isoflourane (Baxter) for whole blood collection from the orbital sinus in EDTA coated tubes and followed by aseptical removal of lung, spleen, and liver.

### Colony-forming units and isolation of cells from the lung

To determine bacterial load, total left lungs were homogenized in MQ+ MGIT™ PANTA™ (BD, 245114) in Gentle MACS M-tubes and plated in 3-fold serial dilution on 7H11 agar (BD, 221870). After 3 weeks of incubation at 37°C, CFU were enumerated.

Lung cells were isolated by digesting total lung in Collagenase VIII (C2139, Sigma) for 30 min. followed by gentle homogenization in Gentle MACS C-tube. Homogenate was forced through 70 μm cell strainer (BD Pharmigen) and incubated in ammonium chloride solution for 5 min to lyse red blood cells. Cells were washed × 2 and resuspended in RPMI + 10% FCS, followed by flow cytometry analysis.

### ELISA

For optimal protein extraction, total left lung homogenate (prepared as described above) was added to 5 × RIPA buffer with protease inhibitors, incubated on ice for 30 min. and centrifuged. Supernatant was collected and ELISA was performed. Quantification of plasma and lung lipocalin-2 was executed by standard ELISA techniques (10, 24). The following antibodies was used: Affinity-purified goat anti-lipocalin-2 (1:500, AF 1857, R&D Systems), biotinylated rabbit anti-lipocalin-2 [1:200, in-house-generated antibody (10)], and horseradish peroxidase Avidin D (1:3,000, 18–4,100, eBioscience). As a surrogate marker of neutrophil influx myeloperoxidase, (MPO) ELISA (ab155458, Abcam) was performed according to manufacturer's instructions.

### RNA and quantitative real time PCR

Right lung lobes and the largest liver lobes were removed and quickly stored in RNA Later (Thermo Fischer Scientific). Organs were homogenized in TRIzol Reagent (Invitrogen) in Gentle MACS M-tubes, and RNA purification was performed according to the manufacturer's instructions. mRNA expression was evaluated by quantitative real-time PCR (qRT-PCR) using the TaqMan system (Applied Biosystems). All samples were analyzed in triplicate. Expression levels of *Actb* and *Gapdh* housekeeping genes were used for internal normalization. Relative gene expression was calculated by the comparative threshold method (25) (2^−ΔΔCt^) and is presented as “fold change of expression” compared to uninfected controls. Results were analyzed using MxPro software (Stratagene). HEX-labeled probes were used for quantification of the internal normalization controls *Actb* (4352341E) and *Gapdh* (435339E). FAM-labeled probes were used for quantification of *Lcn2*: Mm01324470_m1, *Tfrc*: Mm00441941_m1, *Fth1*: Mm00850707_g1, *Slc11a1*: Mm00443045_m1, *Slc11a2*: Mm00435363_m1, *Slc40a1*: Mm0125482, *Bdh2*: Mm00459075_m1, *Lrp2*: Mm01328171_m1, *Slc22a17*: Mm00480680_m1, *Cxcl1*: Mm04207460_m1, *Cxcl2*: Mm00436450_m1, *Ccl5*: Mm01302427_m1, *Cxcl9*: Mm00434946_m1, and *Cxcl10*: Mm00445235_m1. All probes were purchased from Applied Biosystems.

### Multiplex cytokine assay

Bead-based Mouse Magnetic Luminex assay (R&D systems) was performed according to manufacturer's instructions and analyzed by Luminex 200 system. The following cytokines were measured: G-CSF, TNF-α, INF-γ, KC, IL-1β, and MIP2 in total lung homogenate supernatant.

### Flow cytometry

Total B-cell, T-cell, and neutrophil cell counts were estimated by flow cytometry on total lung homogenate 3 and 5 weeks post-challenge using anti-Ly6G APC (clone 1A8, Biolegend), anti-CD11b PE (clone M1/79, Biolegend), anti-CD11c PerCP-Cy5.5 (clone HL3, BD), anti-CD19 APC (clone 1D3, BD), anti-CD4 FITC (clone RM4-5, BD), and anti-CD8 PE (clone 53-6.7, BD). All antibodies were diluted 1:200 from manufacturer stocks. Flow cytometry was performed on BD Accuri C6 and analyzed with FlowJo 10.0.8 software (Tree Star Inc).

### Histopathological analysis

Total lung and heart was removed *en bloc*. Before removal, trachea was catheterized and 1.5 ml of 10% formalin was installed to expand the lungs. In addition, spleen and liver were removed. Organs were fixated in 10% neutral buffered formalin for 24 h and stored in 70% ethanol. Paraffin embedded 5 μm thick sections were stained Ziehl-Neelsen (ZN) and eosin-hematoxylin (H&E).

Immunohistochemically staining for lipocalin-2 was performed on lung sections. Sections were deparaffinized and hydrated. To make epitopes accessible for antibodies, sections were pretreated with proteinase K for 15 min. at 37°C. Endogen peroxidase was blocked with 1% H_2_O_2_. Sections were incubated with 5% rabbit serum for 30 min followed by overnight incubation at 4°C with primary antibody, goat anti mouse lipocalin-2 (1:3,200 AF1857, R&D). Day 2 samples were incubated for 45 min with secondary antibody, rabbit anti goat-HRP (P0449, Dako), and developed with DAB+ (DAB+ Envisions-System K5007, Dako) and counter stained with Mayer's hematoxylin.

Validation of the data was made by two independent pathologists, both blinded of the treatment groups. A semi-quantitative scoring system was used for evaluating the staining results. The slides were scanned using VisionTek digital microscope (Sakura Finetek).

### Statistical analysis

All statistical calculations were performed using GraphPad Prism (GraphPad software v.7.2). CFU, ELISA data, cytokine data, and total cell counts were log-transformed before analysis. Normality was evaluated for group residuals and requirements for parametric analysis were met. Comparison of two groups was performed by students *T*-test with Welch correction, and comparison of more than two groups was performed by one-way ANOVA and Tukey's multiple comparison post-test. When two variables were evaluated (time and genotype), two-way ANOVA with Sidák-Holm post-test was used. Our RT-qPCR data was analyzed by one-way ANOVA applied on ΔCt values. Clinical scores between subgroups were analyzed for determination of dependency with χ^2^-test. Only statistically significant results are depicted on the graphs. *P* ≤ 0.05 was considered significant.

## Results

### Lipocalin-2 competent mice show increased susceptibility to M.tb infection at 3 weeks post-challenge

**Figure 1:**
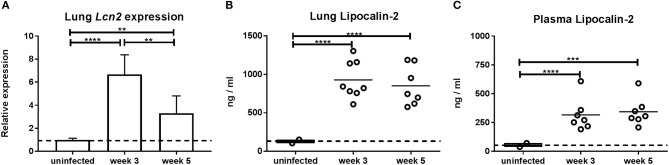
Expression of lipocalin-2 as a function of time after aerosol challenge with M.tb H37Rv. **(A)** Expression of *Lcn2* mRNA in lungs 3 and 5 weeks post-challenge analyzed by qRT-PCR using GAPDH as internal control and presented as fold change (2^−ΔΔCt^). **(B,C)** Lipocalin-2 concentration in lung homogenates **(B)** and plasma **(C)** at 3 and 5 weeks post-challenge. Error bars **(A)** indicate standard deviations and horizontal bars **(B,C)** indicate means. The dotted line indicates mean for uninfected controls. **p < 0.01, ***p < 0.001, and ****p < 0.0001 by ANOVA with Tukey's multiple comparison test.

It has previously been reported that lipocalin-2 is important for control of M.tb during the chronic stage of infection (21). To evaluate the lipocalin-2 expression pattern following M.tb infection, *Lcn2* gene expression was measured in the lungs of WT mice during the growth/primary stage (3 weeks) and the post-peak chronic stage (5 weeks) after aerosol-infection with M.tb relative to that of uninfected WT mice. At 3 weeks post-challenge, we found a 6.6-fold induction of lung *Lcn2* mRNA compared to baseline expression in uninfected mice (Figure 1A). Five weeks post-challenge the lung *Lcn2* mRNA level was still higher, although slightly decreased (3.7-fold higher than in uninfected mice). Similarly, high amounts of lipocalin-2 protein were observed in lungs at 3 and 5 weeks compared to uninfected mice (Figure 1B). The same pattern of lipocalin-2 expression was observed in plasma of the same mice (Figure 1C).

**Figure 2:**
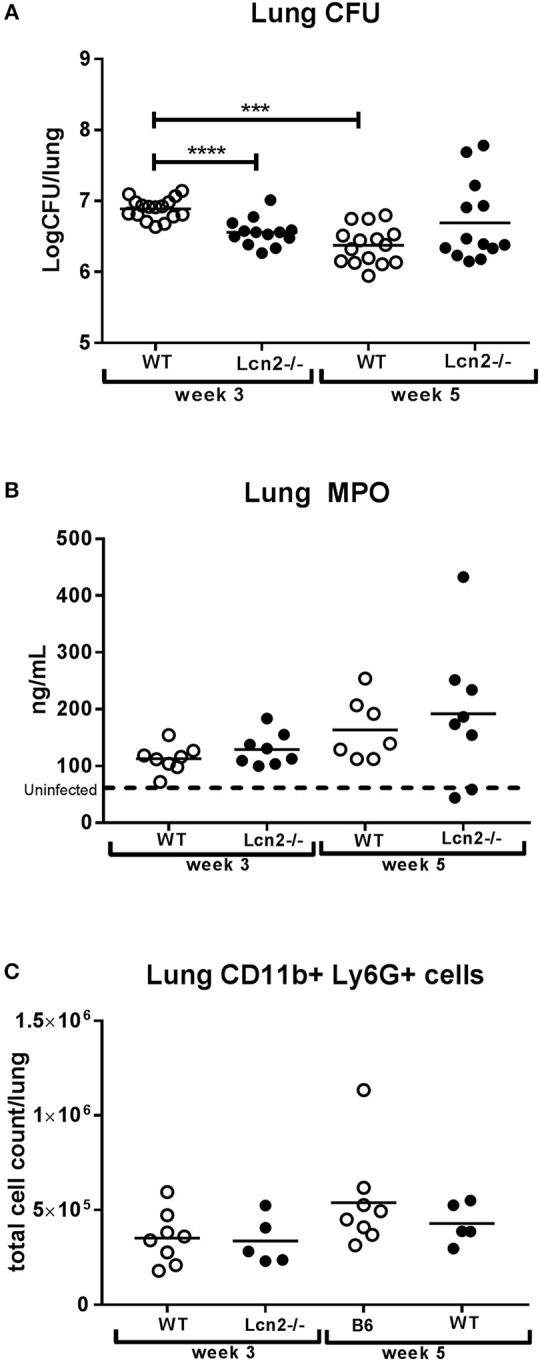
Lack of *Lcn2* expression causes higher infection burden 3 weeks after aerosol challenge with M.tb H37Rv. **(A)** Bacterial load per lung in mice with M.tb H37Rv shown as colony-forming units (CFU) (*n* = 13–16 per group). **(B)** MPO concentrations measured by ELISA in total lung homogenate (*n* = 7–8 per group). **(C)** Total number of Ly6G+, CD11b+ neutrophils isolated from lung measured by flow cytometry (*n* = 5–8 per group). Horizontal bars indicate mean. The dotted line indicates mean for uninfected controls. ***p < 0.001, ****p < 0.0001 by student's *T*-test or two-way ANOVA with Sidaks's multiple comparison test.

High expression of lipocalin-2 was observed at week 3, when formation of lung granulomas is in its early phase. For this reason, we decided to use *Lcn2*^−/−^ mice to examine the importance of lipocalin-2 at this stage where control of M.tb infection is exerted primarily by the innate immune system. To our surprise we observed a nearly 2-fold higher infection burden (CFU, colony-forming units) in lungs of WT mice 3 weeks post-challenge compared to mice unable to produce lipocalin-2 (Figure 2A). At 5 weeks, lung CFU were comparable between WT and *Lcn2*^−/−^. However, when comparing bacterial load in the lungs over time, the bacterial load was reduced in the WT but continuously elevated in the *Lcn2*^−/−^ mice, indicating reduced capability of the *Lcn2*^−/−^ mice in controlling M.tb infection (Figure 2A). The 10-fold lower numbers of mycobacteria in WT mice at week 5 compared to week 3 indicates that WT mice were able to contain the M.tb infection to a level similar to that of *Lcn2*^−/−^ mice.

### Increased susceptibility to M.tb infection in lipocalin-2 competent mice is not related to changes in the inflammatory cytokine response

To determine whether disparities in CFU in the lung were a consequence of differences in neutrophil influx we measured the amount of myeloperoxidase (MPO) in lung lysates. MPO is stored in the primary granules of the neutrophil and is thus a biomarker of the amount of neutrophils in the lungs. No difference in MPO was observed between the two genotypes at either time point (Figure 2B). In addition, we compared the total number of neutrophils in lung tissue by flow cytometry and found no differences in total Ly6G+, CD11b+ cell count between the two genotypes (Figure 2C). These data suggest that the observed differences in mycobacterial loads in the lung were not due to differences in the recruitment of lung neutrophils.

**Figure 3:**
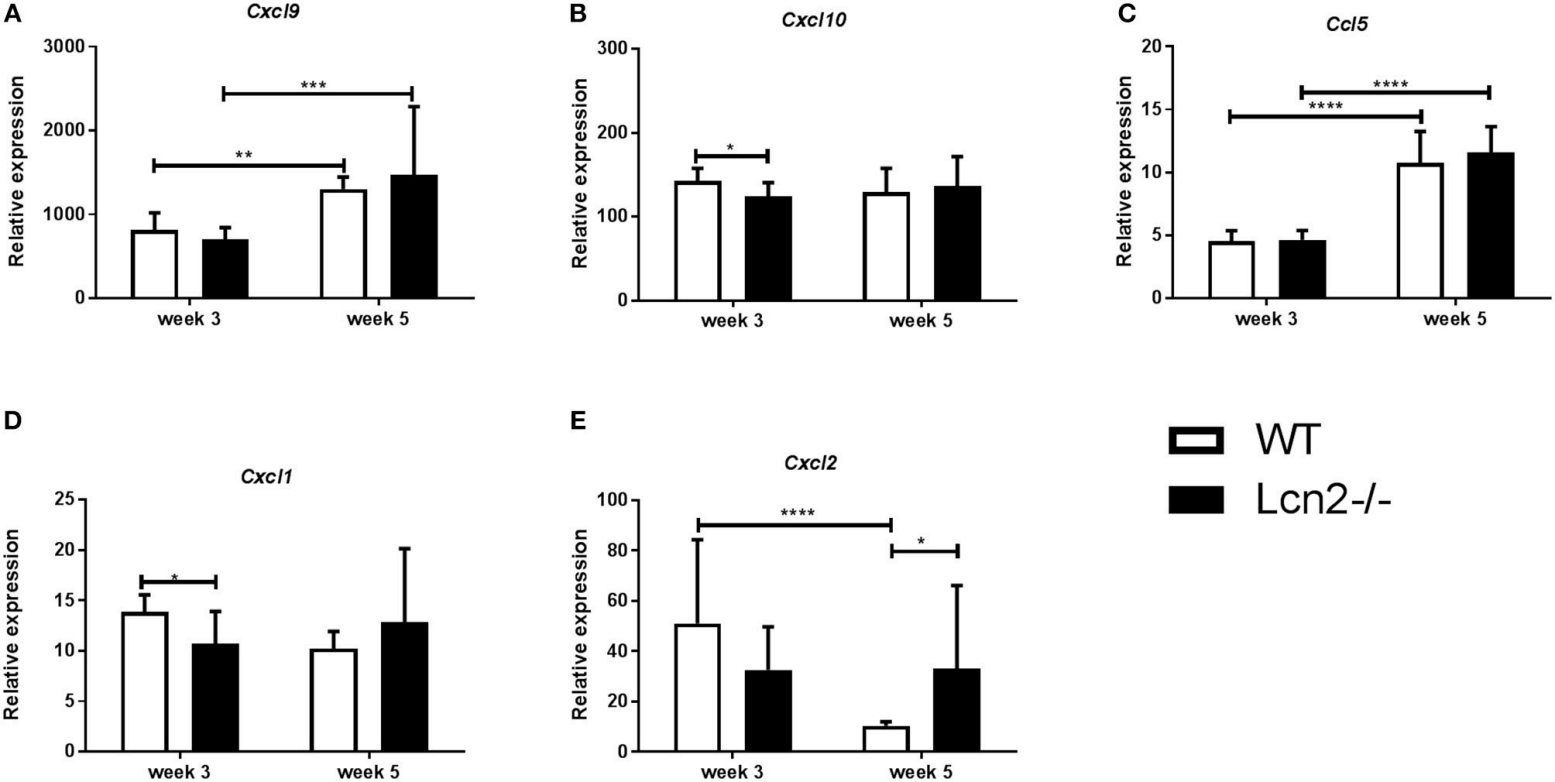
Expression profile in lung 3 and 5 weeks after aerosol challenge with M.tb H37Rv. Relative expression of chemokine mRNAs in lung related to adaptive immune response: **(A)** *Cxcl9*, **(B)** *Ccxl10*, and **(C)** *Ccl5* and innate immune response: **(D)** *Cxcl1* and **(E)** *Cxcl2*. Expression was analyzed by qRT-PCR with β-actin as internal control and normalized to uninfected controls and presented as fold change (2^−ΔΔCt^). (*n* = 7–8 animals in each group). Error bars indicate standard deviations. *p < 0.05, **p < 0.01, and ***p < 0.001, ****p < 0.0001 by student's *T*-test or two-way ANOVA with Sidaks's multiple comparison test.

We wished to examine whether differences in expression of inflammatory response genes that are related to control of M.tb infection (26) could explain the differences in lung CFU between WT and *Lcn2*^−/−^ mice at 3 and 5 weeks post-infection. *Cxcl9* and *Cxcl10* are gamma-interferon induced genes encoding T-cell, NK-cell, and monocyte/macrophage chemoattractants. No major differences were observed in *Cxcl9* and *Cxcl10* transcript levels in lungs between genotypes at 3 or 5 weeks (Figures 3A,B). *Ccl5* (RANTES), encoding another important T-cell activator and chemoattractant, also showed no differences in transcript levels between genotypes (Figure 3C). However, a significant increase in *Cxcl9* and *Ccl5* transcript levels was observed from week 3 to 5 for both genotypes, indicating a mechanism for enhanced recruitment of effector cells related to an adaptive immune response at week 5. *Cxcl1* and *Cxcl2* both encode neutrophil chemoattractants. WT mice had increased *Cxcl1* expression at 3 weeks compared to *Lcn2*^−/−^ mice and *Cxcl2* transcript also tended to be higher in WT mice, however the difference in *Cxcl2* transcript levels between WT and Lcn2^−/−^ mice was not significant (Figure 3D). This indicates that WT mice have increased expression of neutrophil chemoattractant at 3 weeks, which might reflect the increased infection burden in the WT mice at this time point. Conversely, the transcript level of *Cxcl2* was significantly downregulated in WT mice at week 5 compared to week 3, indicating a shift from an innate to an adaptive immune response and better infection control (Figure 3E). This pattern did not apply to *Lcn2*^−/−^ mice, where the *Cxcl2* mRNA levels are unaltered indicating lack of infection control at week 5 compared to WT (Figure 3E). Furthermore, the total number of CD4^+^, CD8^+^, and CD19^+^ cells within the infected lung indicated no difference in the capacity to initiate the adaptive immune response between the two genotypes (data not shown).

### Lipocalin-2 expression in early M.tb infection

Next, we evaluated differences in lipocalin-2 expression and production from the different lipocalin-2-producing cellular compartments, i.e., macrophages/neutrophils and epithelia, in the very early phase of M.tb infection. To this end, chimeric mice were generated by lethally irradiating WT, SJL.WT and *Lcn2*^−/−^ mice followed by transplantation with bone marrow cells from SJL.WT or *Lcn2*^−/−^ mice. Neutrophils and macrophages are the two main hematopoietic cell subtypes that produce lipocalin-2. Neutrophil chimerism in the blood was determined individually for each mouse 7 weeks post-transplantation and was found to be 97–100% donor derived (Supplementary Figure 1). Macrophage chimerism was determined in a pilot experiment by evaluation of BAL fluid 7 weeks post-transplantation and demonstrated 96–99% of all F4/80+ macrophages to be donor-derived (data not shown). The chimeric expression pattern of lipocalin-2 was confirmed by immunohistochemically staining (Supplementary Figure 2).

**Figure 4:**
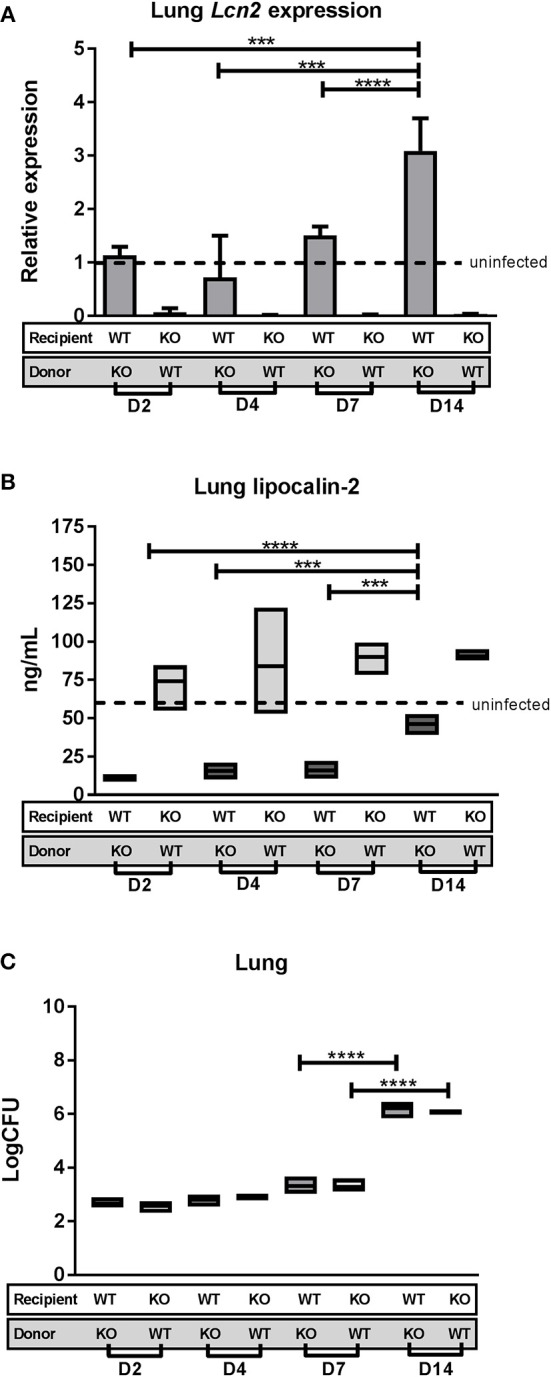
Lipocalin-2 expression profile and CFU in lungs day 0–14 after challenge with M.tb. *Lcn2*^−/−^ mice reconstituted with WT bone marrow (KO/WT) and WT mice reconstituted with *Lcn2*^−/−^ bone marrow (WT/KO) were challenged with M.tb strain H37Rv by aerosol route and euthanized 2, 4, 7, and 14 days post-challenge (*n* = 3–4 in each subgroup). **(A)** Relative expression of *Lcn2* in total lung homogenate analyzed by qRT-PCR with GAPDH as internal control and normalized to uninfected control and presented as fold change (2^−ΔΔCt^). **(B)** Lipocalin-2 concentrations in total lung homogenate measured by ELISA. **(C)** Bacterial load per lung in the two chimeric subgroups shown as CFU. **(B,C)** The boxes indicate the rages of the observations and the horizontal bars indicate mean. Dotted line indicates level for uninfected mice. ***p < 0.001, ****p < 0.0001 by students *T*-test or two-way ANOVA with Sidaks's multiple comparison test. Error bars indicate standard deviations.

*Lcn2*^−/−^ recipient mice reconstituted with SJL.WT bone marrow (KO/WT) and SJL.WT recipient mice reconstituted with *Lcn2*^−/−^ bone marrow (WT/KO), respectively, were challenged with M.tb strain H37Rv through the aerosol route of infection and euthanized 2, 4, 7, and 14 days after infection. Assessment of *Lcn2* transcript and lipocalin-2 protein levels was performed on lung homogenates at the different time points (Figures 4A,B). *Lcn2* transcript in lungs of KO/WT mice was expressed at lower levels than in uninfected WT controls (relative expression = 1) at all time points reflecting the inability of these mice to express *Lcn2* in lung epithelium. For WT/KO mice *Lcn2* expression was comparable to that of uninfected WT mice at days 2, 4, and 7 and increased to an almost 3-fold higher expression level at day 14 post-challenge (Figure 4A).

Lipocalin-2 protein levels in the lung of WT/KO mice were significantly lower at all infection time points compared to uninfected WT controls, demonstrating that the baseline protein level must originate predominantly from circulating neutrophils (Figure 4B). When comparing relative lipocalin-2 levels at the different time points, we saw an increase at day 14, indicating that the contribution of lipocalin-2 from the epithelium is initiated at this time point. Lipocalin-2 levels in the KO/WT mice were comparable to uninfected mice at all time points, illustrating that there is no major contribution of lipocalin-2 from the myeloid population the first 14 days of infection (Figure 4B). Bacterial burden in lungs of the chimeric mice were determined to establish when the differences between genotypes arise during infection. There were no differences in lung CFU between the chimeric groups at these early time-points, with progression of lung infection occurring between day 7 and 14 for both chimeric groups (Figure 4C).

*Lcn2* expression in the liver was also analyzed at the same time points. KO/WT mice had lower expression than uninfected WT controls, while in WT/KO mice the level of *Lcn2* expression was equal to that in uninfected WT controls demonstrating that there was no acute phase response at these early time points (data not shown).

To determine the time of neutrophil influx in lungs, MPO ELISA on lung homogenates was performed. MPO levels were the same as measured in uninfected controls for all time points, indicating that substantial neutrophil migration occurs later than day 14 post-challenge, concomitant with the increase of lung bacterial burden (data not shown). From this we conclude that lipocalin-2 does not play a role in the progress of M.tb infection at time points earlier than day 14 post-challenge.

### Only neutrophil-derived lipocalin-2 leads to increased susceptibility to M.tb

Since lipocalin-2 is released by different cell types present in the infected lung [neutrophils, macrophages, and epithelia cells of the bronchus including alveolar type II pneumocytes (2, 7, 21, 27)] we wanted to explore whether the increased susceptibility to M.tb infection in WT mice at 3 weeks post-challenge was dependent on the origin and location of the produced lipocalin-2. Chimeric mice were challenged with M.tb strain H37Rv by aerosol route of infection 9 weeks post-transplant and assessed 3 weeks post-challenge. This time point was chosen for evaluation of the infected chimeric mice based on the results from the lipocalin-2 expression experiment (Figure 4) where difference in outcome between *Lcn2*^−/−^ and WT occurs between week 2 and 3.

**Figure 5:**
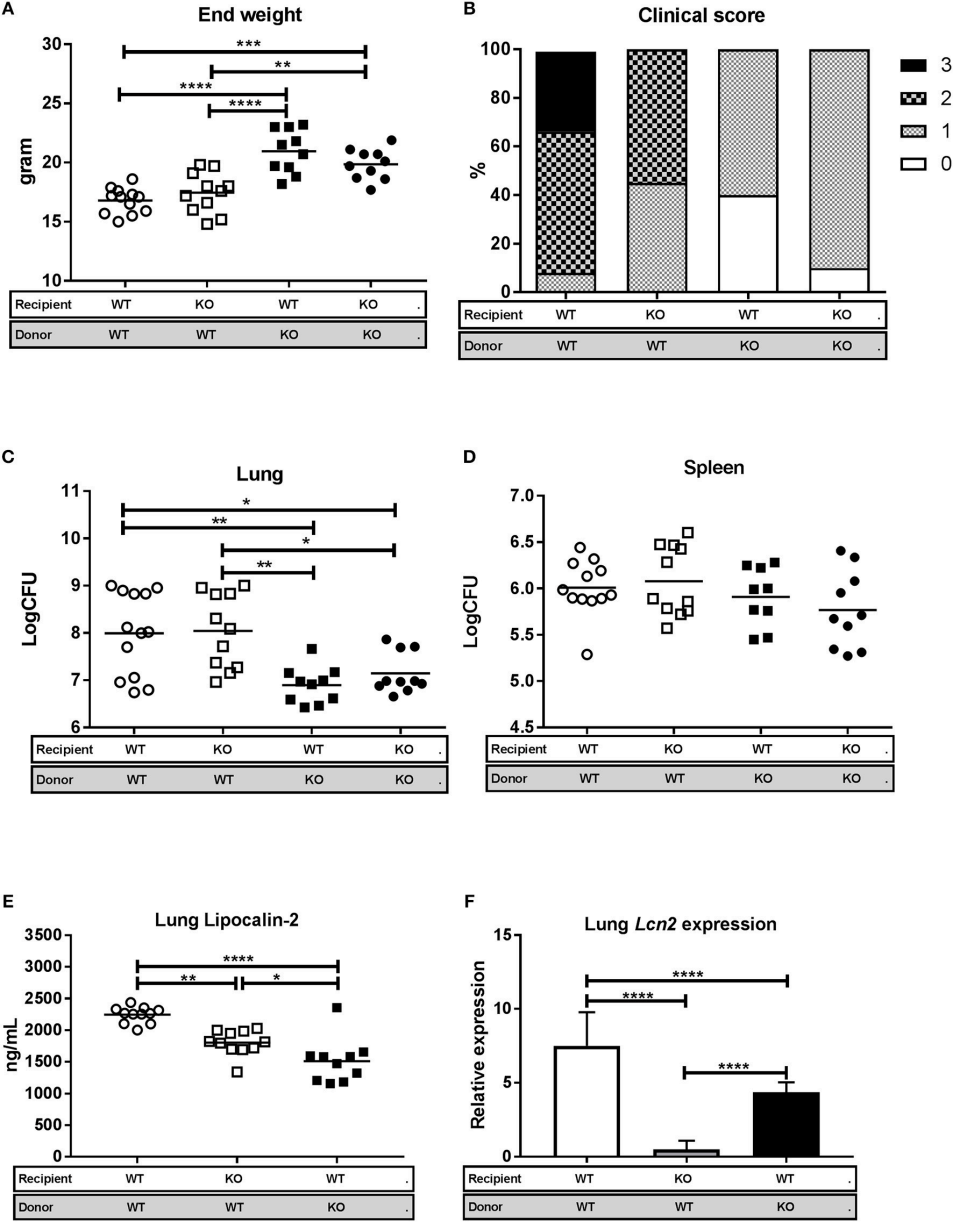
Lipocalin-2 derived from the myeloid compartment is associated with increased susceptibility to M.tb 3 weeks after infection. WT mice reconstituted with WT bone marrow (WT/WT, *n* = 12), *Lcn2*^−/−^ mice reconstituted with WT bone marrow (KO/WT, *n* = 11), WT mice reconstituted with *Lcn2*^−/−^ bone marrow (WT/KO, *n* = 10), and *Lcn2*^−/−^ mice reconstituted with *Lcn2*^−/−^ bone marrow (KO/KO, *n* = 10) were challenged with M.tb strain H37Rv by aerosol route and euthanized 3 weeks post-challenge. **(A)** End weight in grams. **(B)** Clinical severity score of the four chimeric subgroups 3 weeks after challenge (each of the subgroups comprises 100% and the percentage of mice with a specific score is presented by different patterns within the 100%). **(C,D)** Bacterial load per lung **(C)** and spleen **(D)** in the four chimeric subgroups shown as CFU. **(E)** Lipocalin-2 concentrations in total lung homogenate measured by ELISA. **(F)** Relative expression of *Lcn2* in total lung homogenate analyzed by qRT-PCR with GAPDH as internal control and normalized to uninfected control and presented as fold change (2^−ΔΔCt^). Horizontal bars indicate mean. *p < 0.05, **p < 0.01, ***p < 0.001, ****p < 0.0001 by ANOVA with Tukey's multiple comparison test. Error bars indicate standard deviations.

WT recipient mice reconstituted with SJL.WT bone marrow (WT/WT) and *Lcn2*^−/−^ recipient mice reconstituted with SJL.WT bone marrow (KO/WT) showed increased disease progression by clinical evaluation and significant weight loss, compared to SJL.WT recipient mice reconstituted with *Lcn2*^−/−^ bone marrow (WT/KO) and *Lcn2*^−/−^ recipient mice reconstituted with *Lcn2*^−/−^ bone marrow (KO/KO) (Figures 5A,B). The same pattern was observed when comparing lung CFU between the different chimeric subgroups. Both the WT/WT mice and the KO/WT mice had significantly higher bacterial loads in lungs compared to WT/KO and KO/KO mice (Figure 5C). No differences in spleen CFU were seen between groups (Figure 5D). Since there were no differences in clinical score, weight loss, or lung CFU between the WT/WT and KO/WT mice, we conclude that lipocalin-2 from neutrophils and/or macrophages is responsible for the increased susceptibility to M.tb 3 weeks post-challenge and that lipocalin-2 derived from epithelial cells has little importance on outcome.

To further investigate the contribution of lipocalin-2 from the different cellular compartments, i.e., macrophages/neutrophils vs. epithelia, we determined the levels of lipocalin-2 in total lungs of the three chimeric subgroups capable of *Lcn2* expression (Figure 5E). As expected, the amount of lipocalin-2 was highest in the WT/WT mice, but lipocalin-2 from neutrophils and macrophages (KO/WT) as well as lipocalin-2 produced by lung epithelial cells (WT/KO) both contributed to the total amount of lung lipocalin-2.

The increased weight loss, clinical score, and higher lung CFU in the WT/WT and KO/WT suggests that macrophage- and/or neutrophil-derived lipocalin-2 increases susceptibility to M.tb infection 3 weeks after challenge. To investigate whether it was mainly macrophages or neutrophils that contributed to lipocalin-2 in the lung, we performed qRT-PCR on lung homogenate from the chimeric mice (Figure 5F). Lipocalin-2 in neutrophils is exclusively produced during maturation of neutrophil precursors in the bone marrow, so any detected mRNA in the lung will originate from either epithelia or macrophages. The expression of *Lcn2* mRNA in the lungs of KO/WT mice (where any detected *Lcn2* mRNA derives only from macrophages) was slightly lower than uninfected controls, whereas the *Lcn2* transcript levels in WT/KO mice (where *Lcn2* mRNA only represents expression by epithelia) was significantly upregulated demonstrating that *Lcn2* expression from macrophages is negligible under these settings.

**Figure 6:**
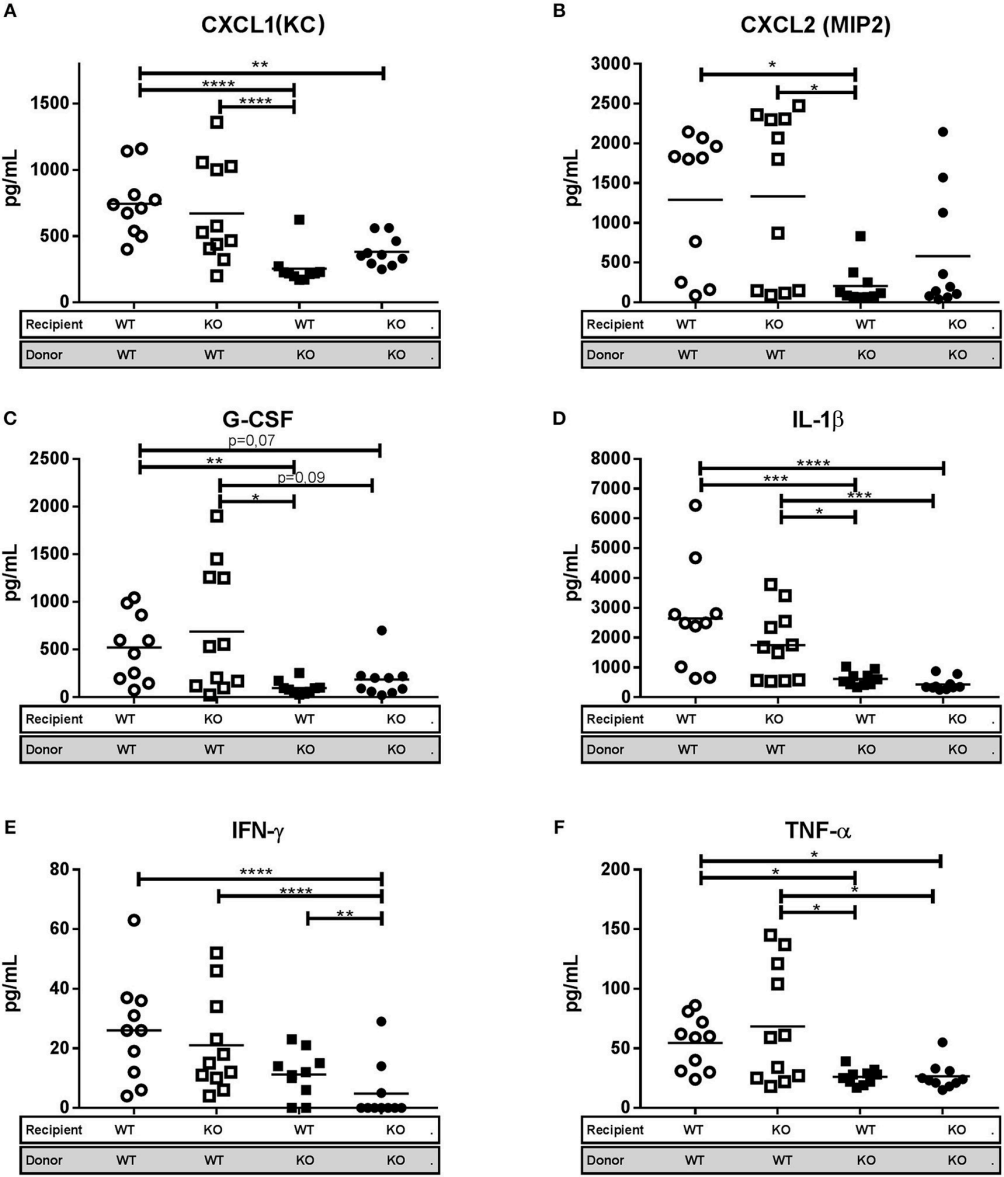
Lipocalin-2 in granulocytes is associated with increased levels of pro-inflammatory cytokines and chemokines in lung. **(A)** Keratinocyte chemoattractant CXCL1 (KC), **(B)** Macrophage inflammatory protein CXCL2 (MIP2). **(C)** Granulocyte colony-stimulating factor (G-CSF), **(D)** Interleukine 1-β (IL-1β), **(E)** Interferon-γ (INF-γ), and **(F)** Tumor necrosis factor-α (TNF-α) protein levels in lung homogenate, from bone marrow transplanted mice 3 weeks post-challenge with M.tb. Horizontal bars indicate mean. *p < 0.05, **p < 0.01, ***p < 0.001, ****p < 0.0001 by ANOVA with Tukey's multiple comparison test. Error bars indicate standard deviations.

To assess whether the inflammatory response differed between the four chimeric subgroups we measured the levels of the neutrophil chemoattractants Cxcl1, Cxcl2, and G-CSF in lung homogenates by multiplex cytokine assay (Luminex). No difference in cytokine levels was observed between WT/WT and KO/WT mice, which both showed a consistent pattern of higher expression levels compared to that in lungs of WT/KO and KO/KO mice (Figures 6A–C). This probably reflects the increased clinical score and higher bacterial burden in WT/WT and KO/WT mice and consequently a more intense inflammatory response. The same pattern of expression was observed for the pro-inflammatory cytokines IL-1β, TNF-α, and IFN-gamma in the four chimeric groups (Figures 6D–F). The increased Cxcl1 and Cxcl2 protein levels in the WT/WT and KO/WT mice corresponds to the observations in Figures 3D,E where WT mice were found to have increased mRNA expression of these two cytokines.

### Evaluation of iron metabolism reveals downregulation of lipocalin-2 receptors

Lipocalin-2 has previously been proposed to play a role in iron homeostasis (28) and we speculated whether the growth-promoting effect of lipocalin-2 at 3 weeks after infections was related to changes in iron metabolism. If release of lipocalin-2 from neutrophils during the earlier stages of M.tb infection (i.e., 3 weeks) could lead to increased accessibility of iron to the infected macrophages, then this could explain the advantage of lacking the lipocalin-2 at this time point. To test this, we quantified the relative expression levels of several genes related to iron homeostasis on total lung homogenate from *Lcn2*^−/−^ and WT mice 3 and 5 weeks post-M.tb challenge.

**Figure 7:**
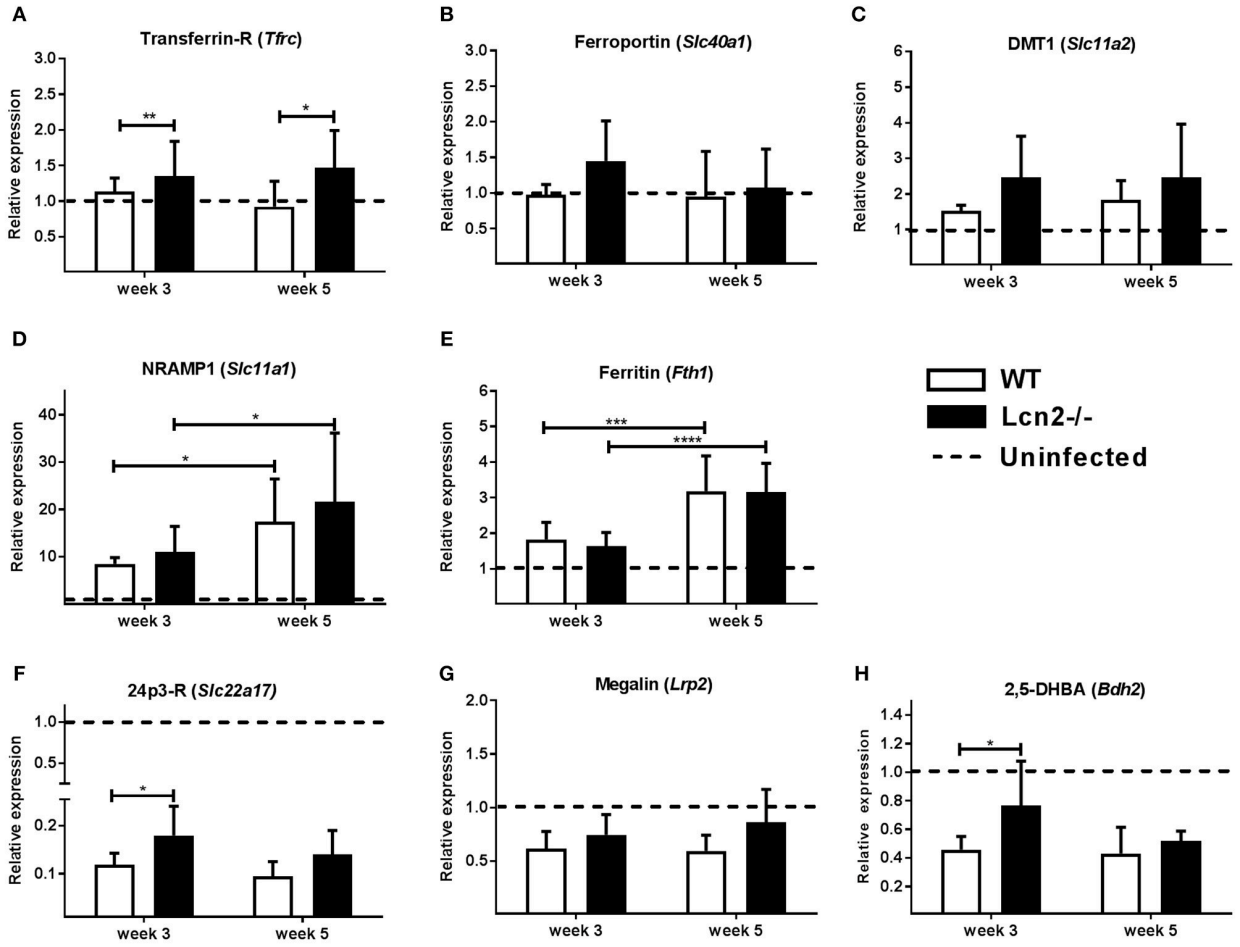
Quantification of transcripts for proteins involved in iron homeostasis in lungs 3 and 5 weeks after aerosol challenge with M.tb H37Rv. Expression of mRNAs related to iron homeostasis in the lung relative to uninfected controls. **(A)** Transferrin receptor (*Tfrc*), **(B)** Ferroportin (*Slc40a1*), **(C)** DMT1 *(Slc11a2)*, **(D)** NRAMP1 (*Slc11a1*), **(E)** Ferritin (*Fth1*), **(F)** 24p3-R (*Slc22a17*), **(G)** Megalin-R (*Lrp2*), and **(H)** 2,5-DBHA (*Bdh2*). Expression was determined by qRT-PCR with β-actin (*Tfrc, Slc40a1, Slc11a1, Slc11a2, Fth1*) or *GAPDH* (*Slc22a17, Lrp2, Bdh2)* as internal controls and normalized to uninfected controls and presented as fold change (2^−ΔΔCt^). (*n* ≥ 7 animals per group). *p < 0.05, **p < 0.01 ***p < 0.001 and ****p < 0.0001 by students *T*-test or two-way ANOVA with Sidak's multiple comparison test. Error bars indicate standard deviations.

Expression of transporters of iron across the cell membrane, transferrin-receptor, ferroportin, and DMT1, were only slightly upregulated compared to uninfected controls (Figures 7A–C). However, transcript levels of transferrin-receptor were higher in *Lcn2*^−/−^ mice at both time-points. NRAMP1 is a phagolysosome-bound protein that pumps iron from the phagocytic vacuole into the surrounding cytosol. There were no differences in expression of NRAMP1 between WT and *Lcn2*^−/−^ mice at the two time-points, but the overall expression was about 10 times higher in infected animals than in uninfected controls at 3 weeks and 15–20 times higher at 5 weeks (Figure 7D). Transcript levels of ferritin were also comparable between genotypes and upregulated 5 weeks post-challenge compared to 3 weeks (Figure 7E). With regard to genes specifically related to lipocalin-2 metabolism, we examined the transcript levels for the two known receptors for lipocalin-2, 24p3-R and megalin (29, 30), which mediate transport of the protein and its bound iron-loaded siderophore into the cell. Transcript levels of 24p3-R in *Lcn2*^−/−^-mice were slightly higher at 3 weeks compared to WT (Figures 7F,G). Interestingly, however, both receptors were significantly downregulated compared to uninfected WT controls. Furthermore, we also looked at the proposed mammalian siderophore 2,5-DHBA, suggested to be a ligand for lipocalin-2 and related to iron homeostasis. Expression of the gene encoding 2,5-DHBA was higher at 3 weeks in the *Lcn2*^−/−^ mice, but also significantly downregulated compared to uninfected controls (Figure 7H). Although the data demonstrates a tendency toward a slightly higher expression of ion transporters in *Lcn2*^−/−^ mice than in WT mice this does not offer an explanation for why WT mice are less efficient in restraining infection at 3 weeks. Indeed, the increased transcript levels of 24p3-R and 2,5-DHBA could reflect an compensatory upregulation in the *Lcn2*-deficient mice at 3 weeks.

### Neutrophils co-localize with M.tb early in infection

**Figure 8:**
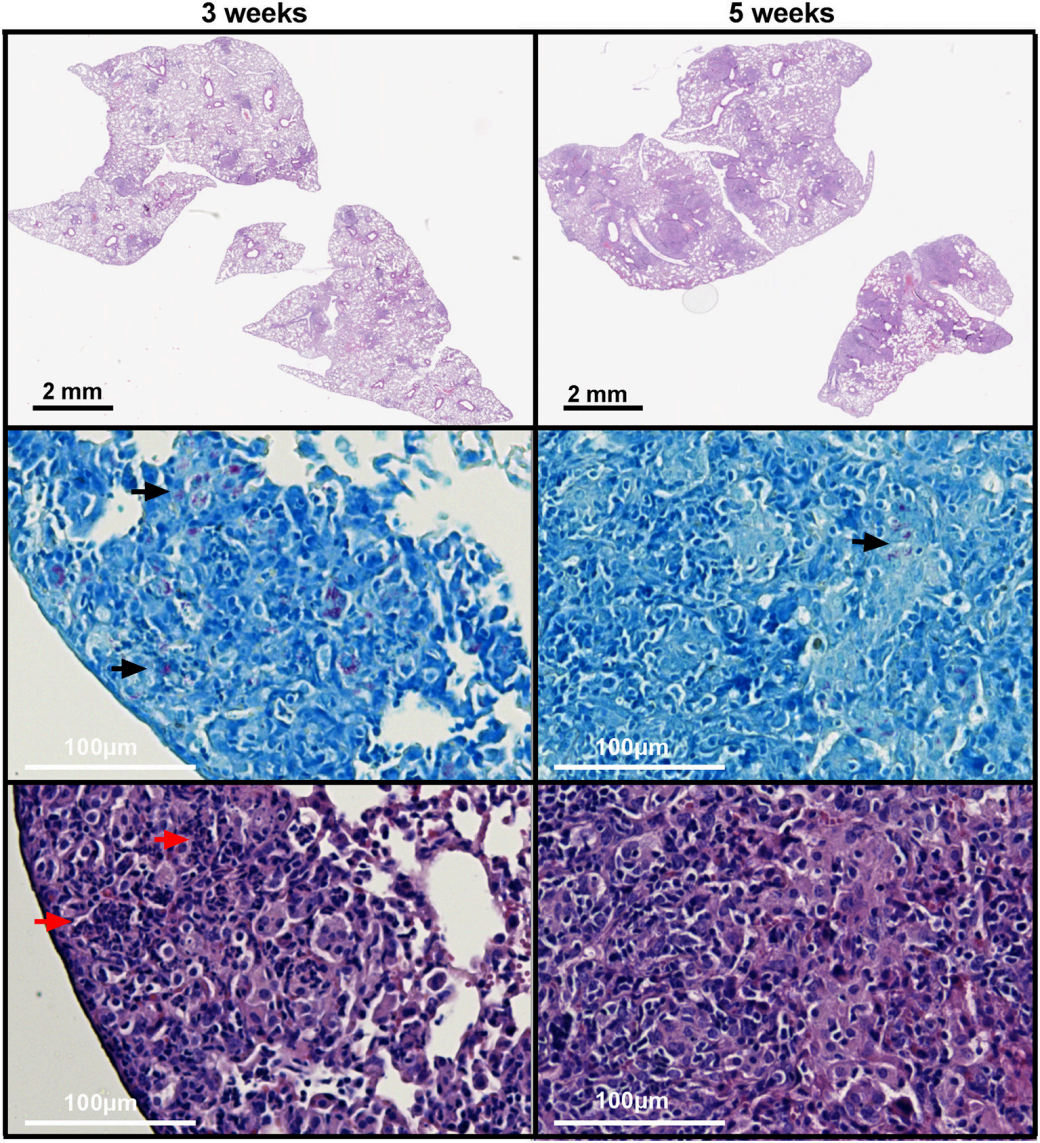
Histopathological features of granulomas in lungs of WT and *Lcn2*^−/−^ mice 3 and 5 weeks after aerosol challenge with M.tb H37Rv. Representative ZN and HE-stained lung tissue from WT mouse 3 and 5 weeks post-challenge with M.tb. Black arrows indicate ZN positive rods and red arrows indicates neutrophils.

As neutrophil-derived lipocalin-2 was responsible for the increased susceptibility in WT mice at 3 weeks post-challenge, we speculated whether this could be explained by closer proximity of neutrophils and mycobacteria at week 3. Histopathological evaluation of lungs revealed that there were no major differences in the structure and histological features between WT and *Lcn2*^−/−^ mice at 3 and 5 weeks, respectively. At 3 weeks post-challenge the granulomas were loosely structured and the inflammatory cells, primarily epithelioid macrophages, lymphocytes, and neutrophil granulocytes, were scattered within the granuloma in close proximity to the mycobacteria. In contrast, at 5 weeks, granulomas were consolidated with a well-organized and confluent outer structure. Mycobacteria were primarily found within the granuloma surrounded by multinucleated giant cells whereas neutrophils were outside the granulomas (Figure 8). Evaluation of neutrophil presence within the granulomas showed increased numbers of neutrophils within the granuloma at 3 weeks compared to 5 weeks. At 5 weeks, only few neutrophils were present, and lymphocytes dominated the cellular rim that encircled the epithelioid center containing the mycobacteria. Granulomas were rather small at 3 weeks, but after 5 weeks they composed 25% or more of the lung tissue. At 5 weeks the presence of mycobacteria within the granuloma was less compared to 3 weeks, indicating that the immune response was controlling the infection (Table 1).

**Table 1:** Semi-quantitative evaluation of lung sections from WT mice and *Lcn2*^−/−^ mice 3 and 5 weeks after aerosol challenge.

**Genotype**	**Weeks post-challenge**	**Granuloma involvement**	**Neutrophil infiltration in granuloma**	**ZN positive mycobacteria in granuloma**	**Granuloma morphology**
WT	3 weeks	1	++	++	Granulomas consist of epithelioid histocytes admixed with neutrophils and lymphocytes. ZN positive mycobacteria are diffusely scattered within the granuloma. The structure of the granuloma is loose without presence of multinucleated giant cells.
		1	++	++
		1	++	+
		1	++	++
		1	++	++
Lcn2^−/−^	3 weeks	1	++	++
		1	+++	++
		1	+++	++
		1	+	+
		1	+++	+
WT	5 weeks	2	++	+	Large consolidated and confluent granulomas with a core of epithelioid histocytes and multinucleated giant cells surrounded by a rim of lymphocytes. Only few neutrophils are present. ZN positive mycobacteria are primarily present within the multinucleated giant cells.
		2	+	+
		3	+	++
		3	+	++
		2	+	+
Lcn2^−/−^	5 weeks	2	+	+
		2	+	+
		1	+	+
		2	+	+
		2	+	++

*Granuloma morphology evaluated in a blinded fashion by a trained pathologist. Degree of granuloma involvement of total lung section: 1: < 25%, 2: 25-50%, 3: >50%. Presence of neutrophils or Ziehl-Nielsen (ZN) positive mycobacteria within the granulomas: +: minor, ++: moderate, +++: substantial*.

## Discussion

Previous studies have revealed the importance of lipocalin-2 in combating M.tb infection. It has been demonstrated that lipocalin-2 inhibits growth of M.tb by sequestration of iron-loaded siderophores in both *in vitro* and *in vivo* models (21–23, 31, 32). Although the innate immune response is primarily important at the early stages of infection, previous *in vivo* tuberculosis studies have focused on the effect of lacking lipocalin-2 at later stages of infection, when the adaptive immune response dominates (33). As lipocalin-2 is an innate immune protein, we found it important to assess its role in M.tb infection at an early time point when formation of the granuloma is incomplete and T-cell mediated immune response is in its early beginning (34).

At 3 weeks post-infection, expression of *Lcn2* mRNA peaked in lungs and high amounts of lipocalin-2 were present in lungs and plasma. Surprisingly, WT mice had significantly higher bacterial load in lungs compared to *Lcn2*^−/−^ mice at this early time point. However, when comparing bacterial load in lungs over time within the two genotypes, WT mice were capable of controlling the infection with a significantly reduced bacterial load at 5 weeks post-infection. *Lcn2*^−/−^ mice, on the other hand, showed comparable bacterial loads at 3 and 5 weeks indicating inability of these mice to restrain the infection. These results emphasize, for the first time, that lipocalin-2 can facilitate M.tb growth at the early phase of bacterial infection.

In a study by Saiga et al. (21) it was found that *Lcn-2* is highly expressed in the lung 2 days after intra-tracheal M.tb installation and thereafter decreased. In our early expression setup (Figure 4), where chimeric KO/WT and WT/KO mice were aerosol infected, we saw no expression of *Lcn2* prior to day 14 after infection and moreover no migration of neutrophils to the lung tissue was observed at these early time points. Following a transient increase of lung neutrophils the day after infection, a major influx of neutrophils to lungs at 3 weeks post-infection was observed also in another M.tb.-infection study (35). This fits our observations, that no major influx of neutrophils takes place before day 14 and supports that 3 weeks was the optimal time point to evaluate the role of lipocalin-2 in innate control of M.tb. The differences between the lipocalin-2 expression profiles in our study and the study by Saiga et al. are likely related to the use of very different infection models. In the Saiga study mice were intratracheally infected with 1 × 10^6^ CFU, leading to a direct installation of a very high dose of bacteria into the lungs. In contrast, we used the more physiological aerosol infection model, where mice inhaled H37Rv-containing droplets, receiving ~1,000 CFU into the lungs, a delivered dose 1,000-fold lower than in the Saiga model. These differences in bacterial load would likely elicit very different innate immune responses in the two scenarios, emphasizing the importance of M.tb model designs.

Previous studies have shown that the importance of cellular source for delivery of lipocalin-2 is dependent on where in the body lipocalin-2 is required for combat of microorganisms (21, 36, 37). In a colitis model, epithelia-derived lipocalin-2 was shown to be the major contributor of protection rather than neutrophil-derived lipocalin-2 (37). In a pneumonia infection model with *Klebsiella pneumoniae*, lipocalin-2 from both epithelium and neutrophils was equally important for full protection against infection (14). Our chimeric animal experiments revealed that only myeloid-derived lipocalin-2 promoted early M.tb infection. Furthermore, by analyzing mRNA expression between the chimeric groups, we found that macrophages did not contribute with any significant amount of lipocalin-2. This indicates that lipocalin-2 derived primarily from infiltrating neutrophils 3 weeks after infection promote the growth of M.tb. Epithelial cells also produce a substantial amount of lipocalin-2, but lipocalin-2 secreted from these cell types does not appear to play a central role in control of M.tb. infection. At 3 weeks post-infection the mycobacteria are primarily located within the lung parenchyma thus rendering the bacteria inaccessible for epithelia-secreted lipocalin-2.

In a study by Guglani et al. (22), using a low dose aerosol M.tb model, they describe a new role for lipocalin-2 in promoting neutrophil influx while also constraining lymphocyte infiltration by regulating *Cxcl9* expression. There was no evidence in our study that lipocalin-2 regulates lymphocytic inflammation early in infection, since gene expression of lymphocytic chemoattractants (*Cxcl9, Cxcl10*, and *Ccl5*) was identical between genotypes. Furthermore, flow cytometry analysis of recruited T- and B-cells showed equal cell counts across the genotypes. Neutrophil migration was also independent of lipocalin-2, given that MPO levels and neutrophil count were the same in WT and *Lcn2*^−/−^ mice. However, the expression profiles of neutrophil chemoattractant *Cxcl2* were different between genotypes, as *Cxcl2* continued to be expressed in the *Lcn2*^−/−^ mice but downregulated over time in WT mice. This probably reflects that the latter mice at 5 weeks post-challenge could control the bacterial infection and thus had a decreased need for further neutrophil influx. Levels of pro-inflammatory cytokines and chemokines showed a matching pattern of expression in the chimeric mice, with higher cytokine/chemokine levels found to be associated with higher bacterial loads in the lungs.

In both WT and *Lcn2*^−/−^ mice a shift from a primarily innate immune gene expression profile at 3 weeks to an adaptive immune profile at 5 weeks was observed for the cytokine data, consistent with the known kinetics of a delayed adaptive immunity to aerosol infection (38, 39), supporting 3 weeks as the optimal time point for evaluating an innate immune response in our model. However, we could not identify any specific changes in the overall immune response that could explain the association between lipocalin-2 and increased susceptibility to M.tb.

In recent years, lipocalin-2 has emerged as an important player in iron homeostasis (28) and we considered whether the growth-promoting effect of lipocalin-2 early in M.tb infection was related to iron metabolism. Indeed, dysregulation of iron uptake in a β-2-microglobulin knockout mouse increases susceptibility to M.tb. growth in macrophages (40). Expression of the three macrophage plasma-membrane related iron transporters; transferrin-R, ferroportin, and DMT1 were only slightly upregulated compared to uninfected mice after M.tb infection. Especially ferroportin was of interest, as overexpression of this cellular iron exporter inhibits intra-macrophage growth of M.tb (41). NRAMP1 is a metal transporter located in the late endosomal and phagosomal membrane of macrophages whose main function is to enhance iron efflux from the phagosome, thereby restricting growth of intracellular pathogens, such as M.tb (42). NRAMP1 was highly upregulated in our model, but expression levels were comparable between the two genotypes.

Of interest, we found the two known receptors of lipocalin-2, megalin and 24p3-R (29, 30), to be excessively downregulated in both infected *Lcn2*^−/−^ and WT mice. It has been proposed that 24p3R can regulate intracellular iron concentration. Holo-24p3 binds 24p3R and is internalized. Its bound iron is released within the cell leading to increased intracellular iron levels (30). The observed decrease in lipocalin-2-binding receptors in our model could illustrate a host defense mechanism that constrains delivery of iron intracellularly, in order to deprive iron from the bacteria.

We also observed a decrease in expression of the suggested mammalian siderophore 2.5-DHBA (*Bdh2*) (43). It has been described that endogenous siderophores can be hijacked by bacteria in order to forage iron and enhance growth (44). *Bdh2*^−/−^ mice showed no increased susceptibility to M.tb in a low dose infection model 150 days post-infection, however, 4 weeks post-infection Bdh2^−/−^ mice had much lower lung CFU compared to WT mice (44). It has been disputed that 2.5-DHBA can mediate lipocalin-2 dependent iron uptake (45), while others have confirmed that 2.5-DHBA can bind lipocalin-2, but the binding affinity is rather low (19). Another suggested mechanism for uptake of lipocalin-2 is binding of iron-loaded catechol by lipocalin-2 followed by megalin-mediated uptake (46). Receptor-independent endocytosis of iron-loaded lipocalin-2 has also been suggested as a mechanism for transport of iron over the cell membrane (47). Together this supports our assumption that early in infection, when innate immunity dominates, M.tb is dependent on intracellular iron delivery from siderophores (both bacterial-produced and mammalian analogs) and lipocalin-2. Yet, their role changes with time as inflammation becomes dominated by the adaptive immune response.

Contrary to earlier beliefs, nascent granulomas promote mycobacterial replication (48). Following exposure to mycobacteria in the lung, macrophages engulf the bacteria and migrate into lung interstitial tissue. Here the macrophages settle and might eventually succumb to the bacterial infection causing the cell to undergo apoptosis. This initiates formation of immature granulomas by recruitment of uninfected macrophages to the site of infection that phagocytose the dead macrophages carrying intracellular mycobacteria. The newly recruited macrophages thereby become infected allowing further mycobacterial expansion (48). This process continues until a mature granuloma with a structured outer layer has formed, coinciding with the onset of the adaptive immune response. In the same manner neutrophils migrate to the immature granuloma and release their content of lipocalin-2, which binds bacterial or mammalian siderophores loaded with iron. We propose a model where infected macrophages take up iron-loaded lipocalin-2 potentially by lipocalin-2 receptor-mediated uptake. Lipocalin-2:siderophore-bound iron may not be delivered directly to the phagocytic vacuole since *in vitro* studies have described that exogenously added lipocalin-2 resides in endosome and do not co-localize with the mycobacterial phagosome (23). Possible mechanisms for release of iron within the cell could be proteolytic degradation of lipocalin-2 following its uptake in the macrophage. Alternatively, iron could be rendered accessible for the bacteria in the macrophage following engulfment and degradation of infected apoptotic macrophages.

The histopathological evaluation demonstrates close proximity of neutrophils and mycobacteria at week 3, but not at week 5, post-challenge. This fits a model where lipocalin-2 at the early stages of infection inadvertently feeds the macrophages with iron essential for mycobacterial growth thus giving the bacteria an initial growth advantage. Following formation of the mature granuloma this is no longer possible as neutrophils are now excluded from direct contact with the infected macrophages, and lipocalin-2 therefore instead exerts an antimicrobial role by binding and clearance of extracellular mycobacterial siderophores following the occasional release of mycobacteria from the mature granulomas. The downregulation of receptors for lipocalin-2 uptake (and production of the mammalian siderophore) following mycobacterial infection could indicate a potential cellular protection mechanism to avoid lipocalin-2-mediated iron uptake in cells invaded by intracellular pathogens.

There are some limitations related to this study. The early effect of lipocalin-2 was only investigated in an H37Rv M.tb infection model. We chose this strain for our experiments since it is the most studied strain with regard to evaluating the role of lipocalin-2 in M.tb infection and we wanted to be able to compare our results with the prior research in this field. It would be of great interest to establish whether the growth promoting effect of lipocalin-2 at the early stages of infection also applies to more virulent clinical M.tb strains. The function of lipocalin-2 in human tuberculosis is almost undescribed. However, lipocalin-2 is described as a potential biomarker of M.tb infection (49) and its importance in infection models with other lung pathogens is well-described. Therefore, our findings in the mouse-model emphasize the importance of illuminating the role of lipocalin-2 in human tuberculosis. When studying lipocalin-2 in human M.tb infection, it is important to keep in mind that human and mouse lipocalin-2 are 85% homologous with regard to amino acid composition and share the same cup-like form with 8 antiparallel β-sheets forming the calyx (28). Transcriptional regulation of lipocalin-2 regulation is however slightly different between humans and mice. Lipocalin-2 is an acute phase protein in mice but not in humans (9, 10), and TNF-α can independently stimulate lipocalin-2 expression in mice, which is not the case in humans (10, 50). Furthermore, mice only produce lipocalin-2 in its monomeric form and it is thus unable to form a heterodimer with MMP-9 (51). These differences in protein structure and transcriptional regulation may influence the biological properties of the protein and cause functional differences between species. A first step could be to establish the function of the human lipocalin-2 ortholog in M.tb mouse models.

In conclusion, this study demonstrates a co-localization of granulocytes and M.tb within the innate granuloma and describes, for the first time, a growth promoting effect of neutrophil-derived lipocalin-2 at the early stages of M.tb infection. We hypothesize that neutrophils deliver lipocalin-2 to mycobacteria-infected macrophages early in infection, thereby increasing the availability of intracellular iron and consequently facilitating mycobacterial growth.

## Ethics statement

Experiments were conducted at Statens Serum Institute's facilities according to institutional guidelines and with permission from the Danish Animal Experiments Inspectorate, license number 2013-15-2934-00859.

## Author contributions

NB, JC, AT, JW, CM, EC, and SD designed research. SD, CL, and EC performed research. SD and PN analyzed the data. SD and JC wrote the paper. All the authors revising it critically for intellectual content including final approval before publication.

### Conflict of interest statement

The authors declare that the research was conducted in the absence of any commercial or financial relationships that could be construed as a potential conflict of interest. The reviewer AT and handling Editor declared their shared affiliation.

## Supplementary material

Supplementary Figure 1 Neutrophil chimerism of transplanted mice included in experiments. **(A)** Gating strategy for estimation of neutrophil chimerism in blood of bone-marrow transplanted mice 7 weeks post-transplant. **(B)** Results presented as the percent of donor derived neutrophils of total neutrophils in bone marrow transplanted mice included in experiments. Data from WT/WT, WT/KO, and KO/WT is presented. KO/KO mice were not tested for chimerism.

Supplementary Figure 2 Immune histochemical staining targeting lipocalin-2 in chimeric mice. Staining for lipocalin-2 of lungs from WT/WT, KO/KO, KO/WT, and WT/KO mice 3 weeks post-challenge with H37Rv M.tb. Pictures illustrate differential expression of lipocalin-2 in epithelial- and myeloid cells depending on chimeric composition.
