# Supplementary figures and images for: Lipocalin-2 Functions as Inhibitor of Innate Resistance to Mycobacterium tuberculosis
Source: Front Immunol. 2018 Nov 26;9:2717. doi: 10.3389/fimmu.2018.02717 (PMC6275245; doi:10.3389/fimmu.2018.02717)

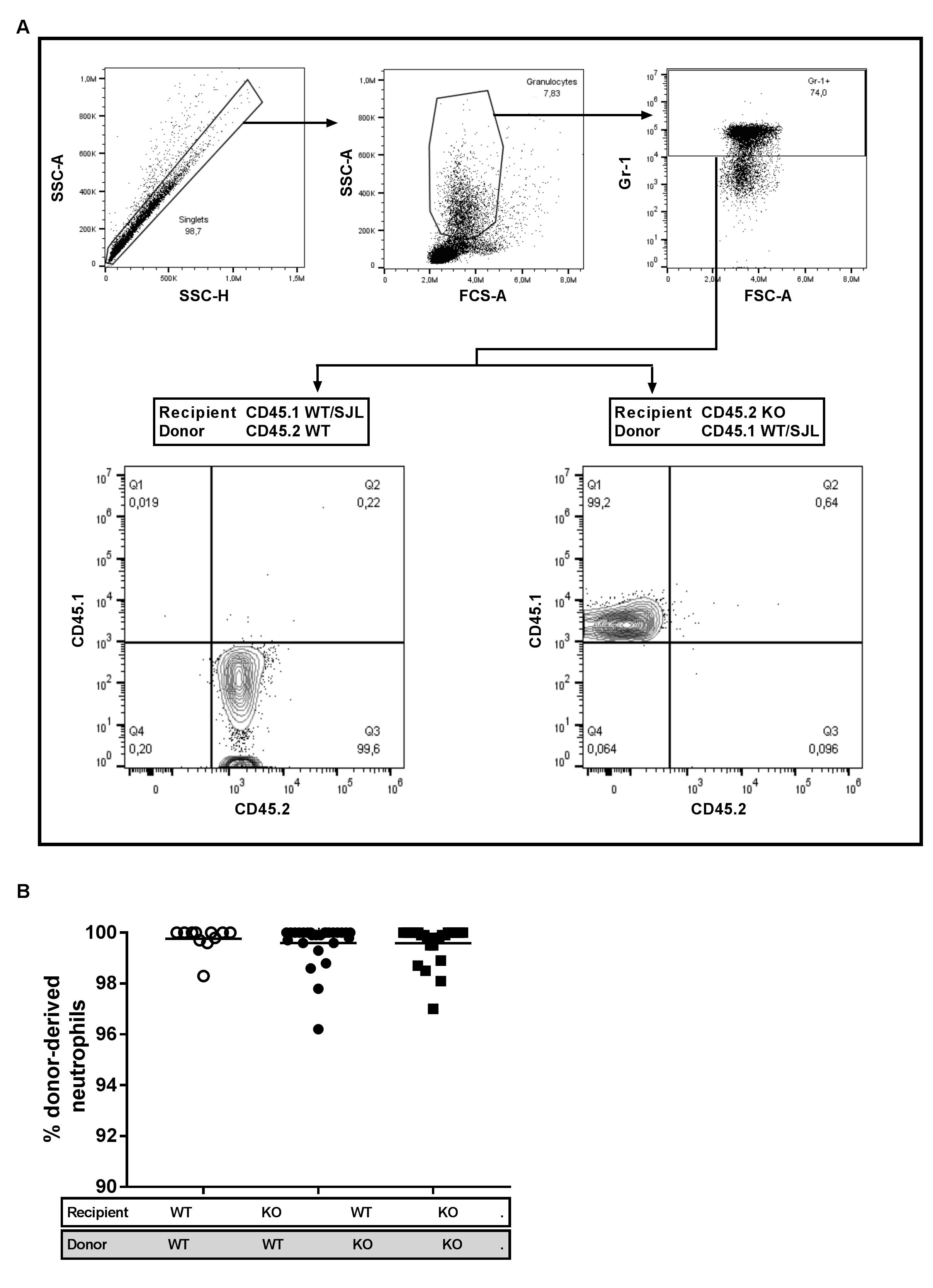

Supplement: Supplementary Figure 1 — Neutrophil chimerism of transplanted mice included in experiments. (A) Gating strategy for estimation of neutrophil chimerism in blood of bone-marrow transplanted mice 7 weeks post-transplant. (B) Results presented as the percent of donor derived neutrophils of total neutrophils in bone marrow transplanted mice included in experiments. Data from WT/WT, WT/KO, and KO/WT is presented. KO/KO mice were not tested for chimerism. [file Image_1.jpg]

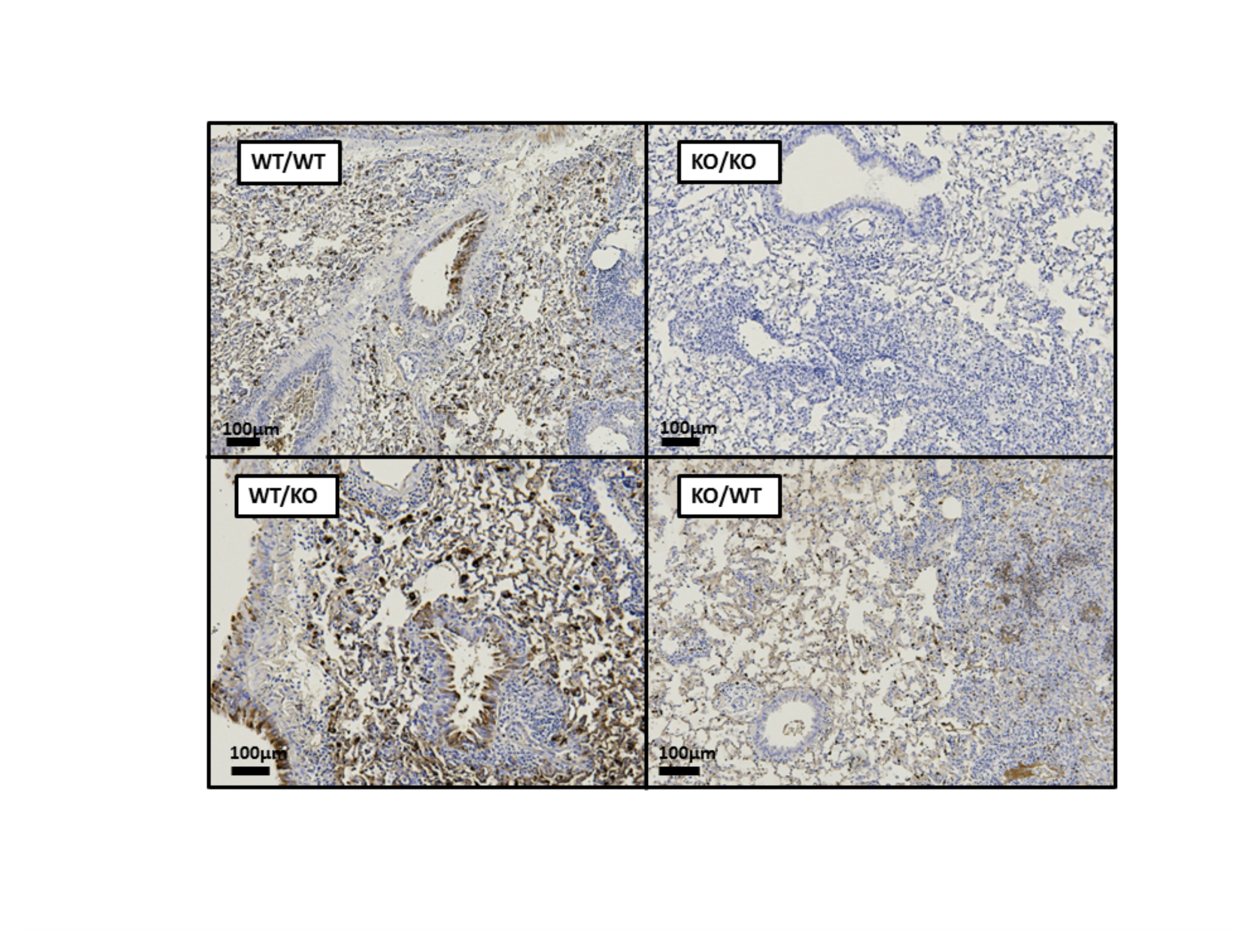

Supplement: Supplementary Figure 2 — Immune histochemical staining targeting lipocalin-2 in chimeric mice. Staining for lipocalin-2 of lungs from WT/WT, KO/KO, KO/WT, and WT/KO mice 3 weeks post-challenge with H37Rv M.tb. Pictures illustrate differential expression of lipocalin-2 in epithelial- and myeloid cells depending on chimeric composition. [file Image_2.TIF]
